# Understanding the cation ordering transition in high-voltage spinel LiNi_0.5_Mn_1.5_O_4_ by doping Li instead of Ni

**DOI:** 10.1038/s41598-017-07139-2

**Published:** 2017-07-27

**Authors:** Junghwa Lee, Nicolas Dupre, Maxim Avdeev, Byoungwoo Kang

**Affiliations:** 1 Department of Materials Science and Engineering, Pohang University of Science and Technology (POSTECH), 77 Cheongam-Ro. Nam-Gu., Pohang, 790-784 Gyeongsangbuk-do South Korea; 2 Institut des Materiaux Jean Rouxel (IMN), Universite de Nantes, CNRS, 2 rue de la Houssiniere, BP 32229, 44322 Nantes Cedex 3, France; 3 Australian Nuclear Science and Technology Organisation, Locked Bag 2001, Kirrawee, DC NSW 2232 Australia; 4 School of Chemistry, The University of Sydney, Sydney, NSW 2006 Australia

## Abstract

We determined how Li doping affects the Ni/Mn ordering in high-voltage spinel LiNi_0.5_Mn_1.5_O_4_(LNMO) by using neutron diffraction, TEM image, electrochemical measurements, and NMR data. The doped Li occupies empty octahedral interstitials (16c site) before the ordering transition, and can move to normal octahedral sites (16d (4b) site) after the transition. This movement strongly affects the Ni/Mn ordering transition because Li at 16c sites blocks the ordering transition pathway and Li at 16d (4b) sites affects electrostatic interactions with transition metals. As a result, Li doping increases in the Ni/Mn disordering without the effect of Mn^3+^ ions even though the Li-doped LNMO undergoes order-disorder transition at 700 °C. Li doping can control the amount of Ni/Mn disordering in the spinel without the negative effect of Mn^3+^ ions on the electrochemical property.

## Introduction

High energy density for lithium ion batteries (LIB) are needed for plug-in hybrid electric vehicles and number of stationary storage applications. With respect to this regard, LiNi_0.5_Mn_1.5_O_4_(LNMO) is a promising cathode material^1–3^ because it has a high redox potential of ~4.7 V, which makes its energy density (650 W∙h/kg) 20% higher than that of conventional LiCoO_2_. Recent studies have shown the promise of Li-ion batteries based on this material, especially composition−structure relationship with electrochemical performance^3–9^. The electrochemical properties of LNMO spinel depend on its structure such as the degree of Ni/Mn ordering^3, 6, 7, 10^ and the presence of Mn^3+^ ions^4, 11^. These two critical factors for electrochemical performance are closely correlated because the disordering of Ni/Mn and the quantity of Mn^3+^ ions are easily coupled by synthesis conditions such as heating temperature, cooling speed, and post-annealing process^6^.

LNMO can have ordered (space group: P4_3_32) and disordered (space group: Fd3m) spinel structures; In ordered spinel, Ni occupies the 4b octahedral sites, and Mn occupies the 12d octahedral sites; in disordered spinel the Ni and Mn are randomly distributed among 16d octahedral sites^1, 12^. Disordered spinel is usually synthesized at 900 °C and thereby has the disordering of Ni/Mn with Mn^3+^ ions due to the loss of oxygen occurring at >700 °C. Additional annealing process at <700 °C transforms the disordered spinel into an ordered spinel because the ordering transition happens at <700 °C. As a result, the ordered spinel has the ordering of Ni/Mn on the two distinct octahedral sites (4b and 12c site) and Mn^4+^ ions by the oxidation of Mn^3+^ ions.

The disordering of Ni/Mn and the presence of Mn^3+^ ions in the disordered spinel strongly affect electrochemical properties. The Mn^3+^ ions can improve electrochemical activity of the spinel because they can increase the electronic conductivity, about two orders of magnitude higher than that of the ordered spinel^6, 13, 14^. However, the presence of Mn^3+^ ions can have a negative effect on the electrochemical properties of the LNMO spinel because Mn^3+^ ions easily disproportionate into Mn^2+^ + Mn^4+^ ions and then Mn^2+^ ions easily dissolve into the electrolyte, especially at high operating potential and elevated temperature^15–17^. Furthermore, disordering of Ni/Mn in the disordered spinel can improve the phase transformation behavior because it extends the solid-solution reaction region on extracting lithium^1, 18, 19^. Considering that two distinct two-phase reactions during charging are underwent, the increase of the solid-solution reaction in the spinel can substantially improve electrochemical activity by reducing mechanical stress/strain induced by lattice mismatches in phases^7^.

Therefore, many efforts have been focused on increasing the Ni/Mn disordering to improve the electrochemical performance of LNMO spinel. The disordering of Ni/Mn can be typically increased by introducing the Mn^3+^ ions that can be controlled by experimental conditions^6, 20^ or that can be controlled by the doping of aliovalent metals instead of Ni because the doping can increase the quantity of Mn^3+^ ions to meet charge neutrality in the spinel^4, 14, 21–23^. Therefore, the LNMO spinel obtained from these approaches always has both the disordering of Ni/Mn and Mn^3+^ ions. Even though the disordered spinel can achieve enhanced electrochemical properties due to facile phase transformation originated from the disordering^1, 8, 18^ and high electronic conductivity originated from Mn^3+^ ions^6, 13^, the presence of Mn^3+^ ions in the spinel can negatively affect electrochemical properties of the spinel such as capacity retention.

Decoupling of Ni/Mn disordering from the Mn^3+^ ions in the spinel can remove this negative effect of Mn^3+^ ions in the LNMO spinel. To achieve this decoupling, the ordering transition during additional annealing process at <700 °C should be suppressed. Recent study reports that the Ni/Mn ordering transition occurs through the formation of Frenkel-type defects that can introduce vacancies in the normal octahedral sites (16d) by displacing Mn and Ni from their normal sites (16d) onto empty octahedral interstitials site (16c) by combining DFT calculation with *in-situ* TEM measurement. The formation of Frenkel-type defects can be a main limiting step for the ordering transition because of its large activation barrier^24^. Taking the ordering transition pathway into account, there can be several ways to increase the Ni/Mn disordering. For example, the approaches that are based on the use of Mn^3+^ ions can change electrostatic interactions between metals in normal 16d sites due to different oxidation state and different interatomic distance in the disordered spinel structure resulting in the increase of Ni/Mn disordering.

Recently, we reported that Li doping to replace Ni in the spinel can decouple the Ni/Mn disordering from the presence of Mn^3+^ ions and the Li doped spinel achieves superior electrochemical performance even without the effect of Mn^3+^ ions on the electrochemical property. Li doping combined with a long additional annealing at 700 °C of the LNMO spinel increased the disordering of Ni/Mn but minimized the quantity of Mn^3+^ ions. Understanding how the doped Li in the spinel affects the Ni/Mn ordering transition is very important, because controlling the Ni/Mn ordering transition pathway presents a new way to increase Ni/Mn disordering without introducing Mn^3+^ ions. In this study, we show how doped Li in spinel affects the Ni/Mn ordering transition by using NMR, neutron powder diffraction (NPD), TEM, and electrochemical measurements. The Li occupies empty octahedral interstitials (16c sites) before the ordering transition, and move to the normal octahedral sites (4b site in P4_3_32) after the transition. Occupation of these sites by Li strongly impedes the Ni/Mn ordering transition. As a result, the Li-doped sample maintained disordering of Ni/Mn even after the Ni/Mn ordering transition step; the Li doping also minimized the quantity of Mn^3+^ ions. Controlling the Ni/Mn ordering transition pathway is an effective way to control the amount of Ni/Mn disordering in the spinel without involving Mn^3+^ ions.

## Results

### Control the Ni/Mn disordering in LNMO spinel without the effect of Mn^3+^ ions by doping Li instead of Ni

**Figure 1:**
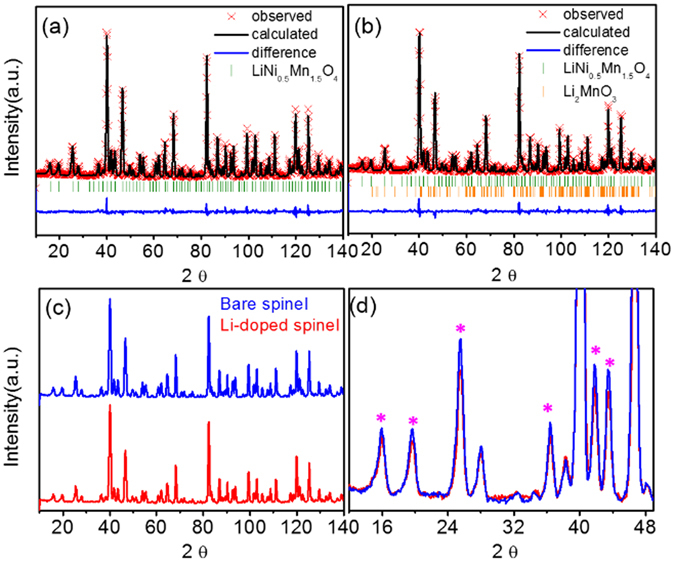
Representative refinement profiles of powder neutron diffraction (ND) of (**a**) bare −700 °C sample and (**b**) Li-doped −700 °C sample. The Bragg peak positions are for LNMO spinel (green bare) and for Li_2_MnO_3_ (orange bar) (**c**) comparison the ***Neutron diffraction*** and (**d**) cthe superstructure peak of bare −700 °C sample and Li-doped −700 °C sample (diffraction peaks corresponding green: LNMO spinel, orange: Li_2_MnO_3_).

LiNi_0.5_Mn_1.5_O_4_ (bare sample) and Li_1.1_Ni_0.45_Mn_1.5_O_4_ (Li-doped sample) show similar neutron diffraction (ND) patterns (Fig. 1) for the 700 °C (ordered) samples (after a 2^nd^ heat-treatment at 700 °C for 48 h). The Rietveld refinement based on P4_3_32 space group was performed using the FullProf software, Table 1 summarized the results of the refinements. And the detailed refined parameters and the agreement factor of Rietveld refinement results is summarized in Table S1. These two samples had similar lattice parameter: 8.171 Å for the bare −700 °C sample and 8.173 Å for the Li-doped −700 °C sample, which are quite similar with lattice parameter of 700 °C spinel with a P4_3_32 space group^1, 18^. The comparison of lattice parameter of the 700 °C samples indicates that the 2^nd^ heat-treatment of the samples lead to similar amount of Mn^3+^ ions as already reported in the literature. Reduced lattice parameters in the 700 °C samples than 900°C (disordered) samples^1, 18^ could be explained by the oxidation of Mn^3+^ to Mn^4+^ through taking oxygen during the 2^nd^ heat-treatment. This indicates that the 700 °C samples have negligible content of Mn^3+^ ions.

**Table 1:** NPD data based Rietveld refinement results of the bare −700 °C and the Li-doped −700 °C.

Sample	Wt. fraction		Space group	Lattice parameter	Site occupancy		
	LiNi_0.5_Mn_1.5_O_4_	Li_2_MnO_3_			Mn/Ni (12d)	Ni/Mn (4b)	Li (4b)
Bare −700 °C	1.0	—	P4_3_32	8.171(1)	1.487/0.013	0.487/0.013	0
Li-doped −700 °C	0.981(1.13)	0.019(0.21)	P4_3_32	8.173(2)	1.463/0.037	0.432/0.037	0.067(3)

Neither 700 °C samples included rock-salt related secondary phases such as NiO or Li_x_Ni_y_O, whereas the Li-doped samples had a trace of ~2 wt% Li_2_MnO_3_ but the bare −700 °C sample did not. Both 700 °C samples show additional peaks around 24° and 42° (Fig. 1c) but the intensity of these additional peaks in the Li-doped −700 °C sample is much weaker than in the bare −700 °C sample. Given these additional peaks originate from the degree of Ni and Mn ordering in the two octahedral sites, 4b site for Ni and 12c site for Mn in the ordered structure^1^, the degree of Ni/Mn ordering in the Li-doped −700 °C sample could be lower than that in the bare −700 °C sample. It should be emphasized that the Li-doped −700 °C sample had weaker additional peaks than the bare −700 °C sample even though the two samples underwent the same 2^nd^ heat-treatment at 700 °C for 48 h that is well known as the transition condition of Ni/Mn ordering. It should be noted that the refinement analysis of the 700 °C samples is carried out with a P4_3_32 space group even though the Li-doped −700 °C sample has the increased portion of the Ni/Mn disordering.

Also atomic occupancy for both 700 °C samples can be refined by FullProf program^6, 12^ because the neutron scattering contrast between Ni, Mn and Li atoms is very high with their coherent scattering lengths being 10.3, −3.73 and −1.9 fm, respectively. So, we tried to refine the occupancy of doped Li in 4b and Ni/Mn in 4b, 12d. In contrast to the bare −700 °C sample, the analysis of the NPD data for the Li-doped −700 °C sample clearly indicated higher Ni/Mn local disordering than [Li_1.0_]_8a_[Ni_0.487_Mn_0.013_]_4b_[Mn_1.487_Ni_0.013_]_12d_O_4_ (bare sample) and the presence of Li on the (4b) site yielding the formula [Li_1.0_]_8a_[Li_0.067_Ni_0.432_Mn_0.037_]_4b_[Mn_1.463_Ni_0.037_]_12d_O_4_ (Li-doped sample), which represents a slight excess of Li content (~6.7%) in Ni site(4b) with respect to the theoretically formulated amount. Given that the Li-doped sample already has Li_2_MnO_3_ as an secondary phase as shown in Table 1, the solubility limit of the Li into the spinel structure can be conservatively estimated at 6% based on NPD. Furthermore, as the doping amount of Li into LNMO spinel increases, the portion of Li_2_MnO_3_ has significantly increased with decreased LNMO spinel portion leading to the formation of a layered-spinel composite (in Figure S2).

**Figure 2:**
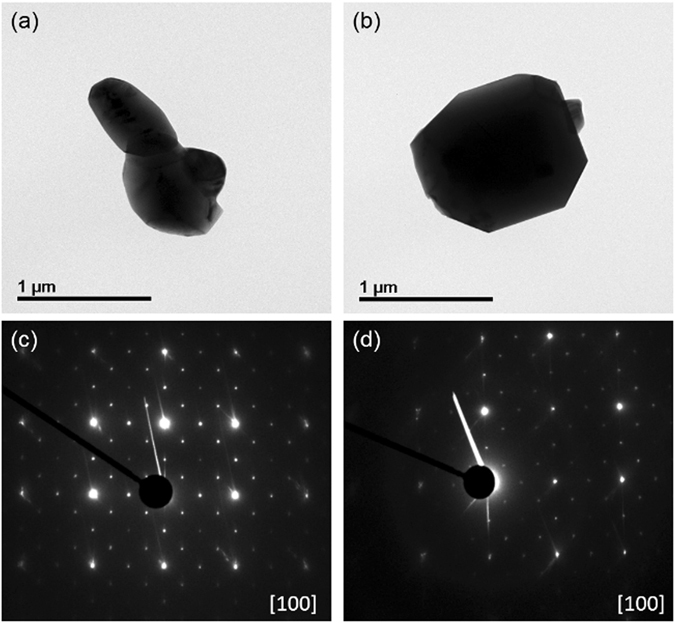
TEM images of (**a**) bare −700 °C sample (**b**) Li-doped −700 °C sample. Selected area electron diffraction patterns of (**c**) bare −700 °C sample (**d**) Li-doped −700 °C sample at zone axis of [100].

Furthermore, the electron diffraction patterns obtained from the two samples in Fig. 2c and d clearly demonstrate that both samples have a little different local cation ordering structure. The bare −700 °C sample exhibited strong additional spots in Fig. 2c indicating that the Ni and Mn ions in ordered spinel structure are locally well ordered in [100] zone^8^. And the Li-doped −700 °C sample showed similar electron diffraction pattern at zone axis of [100] with the bare −700 °C sample corresponding to the ordering of Ni and Mn in the Li-doped −700 °C sample. However, the Li-doped −700 °C sample shows much weaker additional spots than the bare −700 °C sample. The magnitude of this diffraction intensity is directly related to a structure factor, which is related with atom densities at the plane. Therefore, weaker additional diffraction peak intensity of the Li-doped −700 °C sample means that the Ni/Mn local ordering is not well developed even with the ordered (P4_3_32) structure. The lower intensity of the additional spots is in good accordance with the low intensity of superstructure peaks in the NPD data in Fig. 1. In the Li-doped −700 °C sample, the long-range ordering of the Ni/Mn is similar to the ordered structure while the Ni and Mn ions are locally disordered. Based on these structural characteristics, the Li-doped −700 °C sample had increased the Ni/Mn disordering without involving Mn^3+^ ions even though it was subjected to the 2^nd^ heat-treatment at 700 °C for 48 h which typically results in a Ni/Mn ordered spinel.

**Figure 3:**
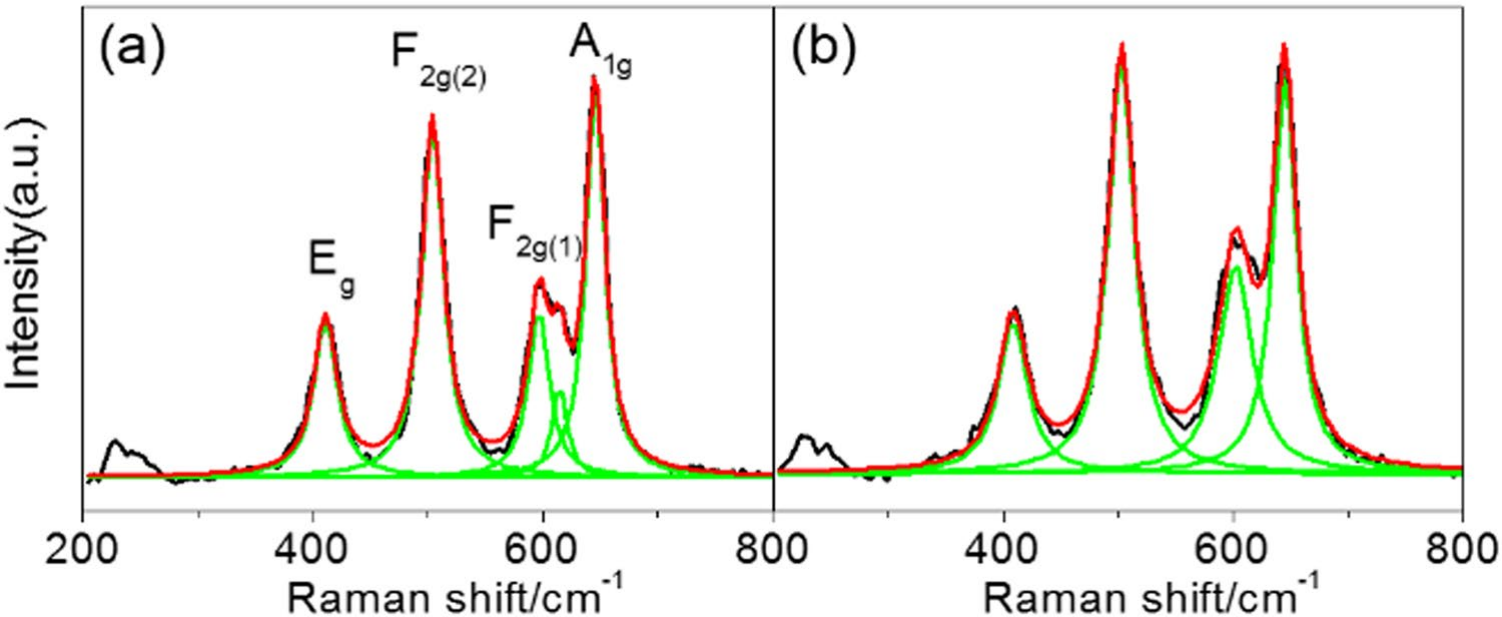
Deconvolution Raman spectra of (**a**) bare −700 °C sample (**b**) Li-doped −700 °C sample.

Both samples were additionally characterized by Raman spectroscopy as shown in Fig. 3. Raman spectroscopy is useful tool to investigate Ni/Mn ordering in spinel due to sensitive enough to the crystal symmetry^5, 25^. The strong signal of A1g band at 633 cm^−1^ and the split of the F_2g(1)_ band at 593 and 612 cm^−1^ are ascribed to the symmetric Mn-O stretching vibration and the peak around 390 and 482 cm^−1^ can be assigned to the Ni–O stretching mode. The degree of Ni/Mn disordering depends on the splitting tendency of the F_2g(1)_ band at 593 and 612 cm^−1^ and broadness of Raman bands. The Li-doped −700 °C sample shows negligible splitting of the F_2g(1)_ with broad Raman bands whereas the bare −700 °C sample shows the splitting of the F_2g(1)_ with narrow Raman bands. Therefore, the features of Raman spectra in the Li-doped −700 °C sample are similar to those in the bare-900 °C sample (in Figure S3, Table S2). This similarity clearly indicates that the Li-doped −700 °C sample has strong Ni/Mn disordering tendency compared to the bare −700 °C sample. In Table 2, the FWHM of those peaks clearly show that the Li-doped −700 °C sample has increased the broadness of those peaks and splitting tendency of the F_2g(1)_ band are decreased compared to the bare −700 °C sample^13^. As a result, the Li-doped −700 °C sample can have larger disordering of Ni/Mn than the bare −700 °C sample even with the 2^nd^ annealing at 700 °C for 48 h^1^.

**Table 2:** Peak designation and FWHM of deconvoluted Raman spectra of the (a) bare −700 °C sample and (b) Li-doped −700 °C sample.

Peak designation	FWHM	
	(a) Bare −700 °C	(b) Li-doped −700 °C
A_1g_	19.4846	24.8280
F_2g(1)_	22.2156	35.2368
F_2g(2)_	23.1244	27.5320
E_g_	24.8865	30.7332

Magnetic measurements can also be used to qualitatively determine the degree of cation ordering^26–28^. Figure S4 shows the temperature dependence of the magnetic susceptibility (left axis) and reciprocal magnetic susceptibility (right axis) for both samples. LiMn_1.5_Ni_0.5_O_4_ spinel undergoes a ferromagnetic (FM) ordering transition at T_c_ ∼120 K. The FM transition temperature (T_c_) for the Li-doped −700 °C sample is lower than that for the bare −700 °C sample by 6 K. Such a variation T_c_ reflects the degree of Ni/Mn ordering in LiMn_1.5_Ni_0.5_O_4_ spinel. Given that the transition temperature between paramagnetic and ferromagnetic will be higher for more atomically ordered samples, the lowering of T_c_ in the Li-doped −700 °C sample can show the increase of Ni/Mn disordering in LiMn_1.5_Ni_0.5_O_4_ spinel^12, 26^. And both samples have similar effective magnetic moments (µ_eff_) which correlates to the average oxidation state of Ni and Mn ions^26, 29^. It means that both samples have similar oxidation state of Ni and Mn ions. The estimated T_c_ and µ_eff_ for each sample are listed in Table S3. These magnetic properties of the two samples are consistent with other structural characteristics. The combination of various techniques clearly demonstrates that the Li doping instead of Ni in high voltage spinel can affect the Ni/Mn ordering transition behaviors leading to the increase of the Ni/Mn disordering without the effect of Mn^3+^ ions on the ordering transition.

**Figure 4:**
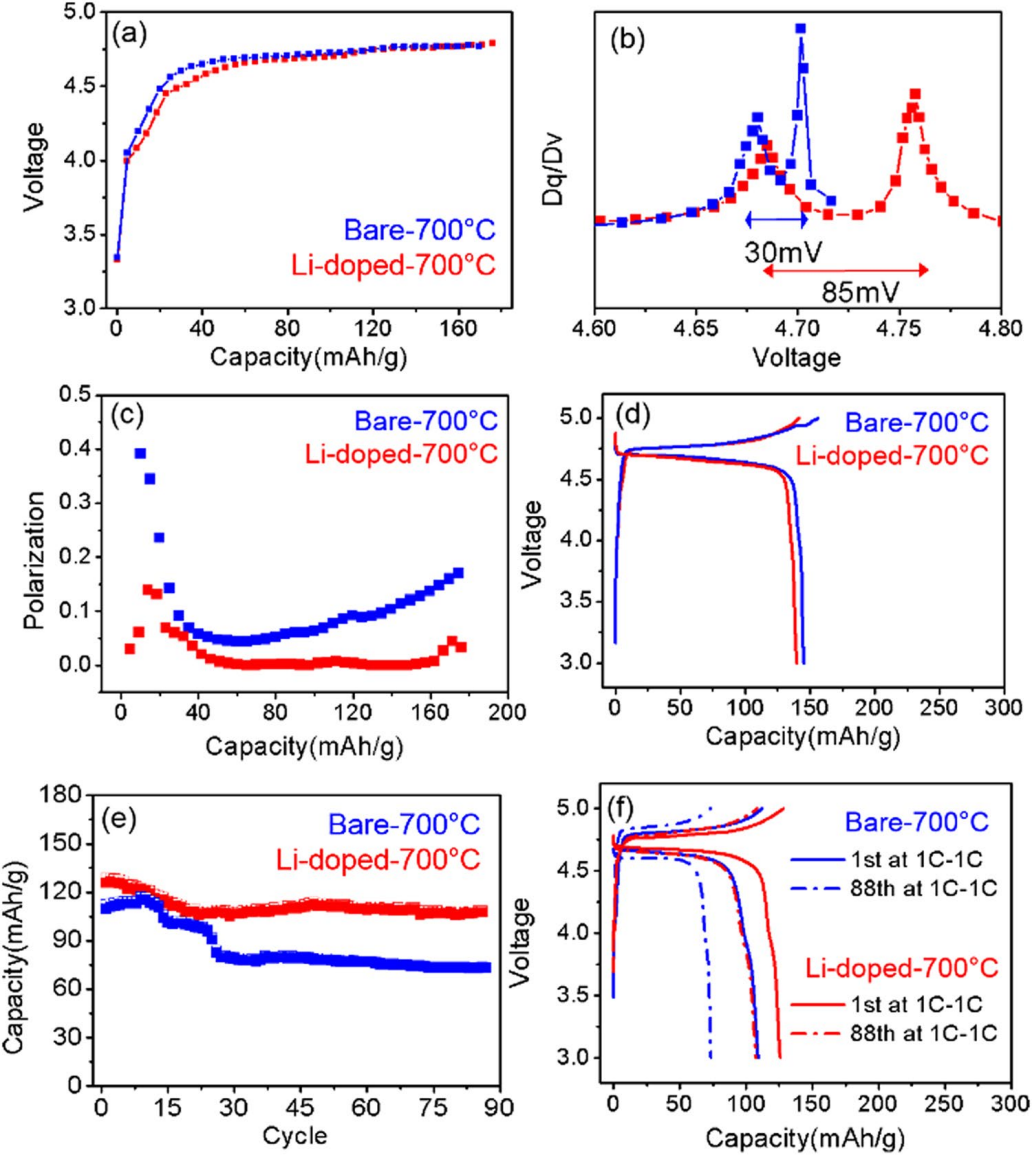
(**a**) Open circuit voltage (OCV) profiles from GITT measurement (**b**) Differential capacity plot (dV/dQ) of the bare −700 °C and the Li-doped −700 °C sample (**c**) Polarization during charging (**d**) Voltage curve at C/5 rate and (**e**) Capacity retention (**f**) Voltage curves at 1C-1C rate for the bare −700 °C sample and the Li-doped −700 °C samples.

The increase of Ni/Mn disordering in the Li-doped −700 °C sample was also confirmed in electrochemical properties by the voltage gap between the Ni^2+^/Ni^3+^ and Ni^3+^/Ni^4+^ redox potentials during the charging process compared to that of the bare −700 °C sample. GITT measurements of the two samples during the charge process in Fig. 4 clearly show their different electrochemical properties. The Li-doped −700 °C sample has larger voltage gap than the bare −700 °C sample as shown in the differential capacity plot (dQ/dV) in Fig. 4b. The voltage gap increased from 30 mV in the bare −700 °C sample to ~85 mV in the Li-doped −700 °C sample. Considering that the voltage gap is correlated with the degree of Ni/Mn disordering^30^, the increased voltage gap indicates that the degree of Ni/Mn disordering is greater in the Li-doped −700 °C sample than in the bare −700 °C sample even though the two samples were prepared in the same experimental condition. Furthermore, the polarization in Fig. 4c significantly reduced in the Li-doped −700 °C sample during the delithiation process compared to the bare −700 °C sample. Low polarization in the Li-doped −700 °C sample can result from the increase of the solid-solution phase reaction that can be caused by the increase of Ni/Mn disordering^5^.

From the voltage curve at C/5 in Fig. 4d, lower capacity of the Li-doped −700 °C sample than the bare −700 °C sample can be a result of the decreased Ni^2+/4+^ redox reaction by reduced Ni quantity. Furthermore, the Li-doped −700 °C sample and the bare −700 °C sample barely have the redox reaction at ~4.0 V that originates from the redox reaction of Mn^3+/4+^. It clearly indicates that the quantity of Mn^3+^ ions in the two samples annealed at 700 °C is negligible. This is also consistent with X-ray Absorption Near-Edge Spectroscopy (XANES) data of the samples in Figure S5. These electrochemical observations such as the increase of Ni/Mn disordering and negligible content of Mn^3+^ ions in the Li-doped sample are well consistent with the structural and magnetic characteristics. As a result, doped Li instead of Ni can suppress the Ni/Mn ordering transition that usually occurs during the 2^nd^ heat-treatment process resulting in the increase of Ni/Mn disordering and in a negligible amount of Mn^3+^ ions.

To estimate the cycling stability of both samples, a 1 C rate (take 1 h to fully charge/discharge) is used for both charge and discharge in Fig. 4e,f. The reversible capacity of the Li-doped sample is 127 mAh/g, which is slightly higher than the bare −700 °C sample (~110 mAh/g). Also, the capacity retention of the Li-doped −700 °C sample (87%) is higher than for the bare −700 °C sample (66%) after 88 cycles, suggesting that the Li-doped −700 °C sample has an improved electrochemical performance. Also we recently demonstrate that the rate capability of the Li-doped −700 °C sample was substantially improved compared to that of the bare −700 °C sample and can strongly depend on the amount of Ni/Mn disordering in the spinel^5^. Especially, the Li-doped −700 °C sample has very high discharge rate capability even though it barely has Mn^3+^ ions in Figure S6 ^5^. Even at 58 C rate (~1 min discharge), the Li-doped −700 °C sample achieved 60 mA∙h/g, which is 44% of theoretical capacity whereas the bare −700 °C sample did not show any measurable capacity at >40C discharge rate in Figure S6.

### The effect of the doping Li on the degree of Ni/Mn ordering

#### Disordering of Ni/Mn in the Li-doped −700 °C and the bare −700 °C sample

**Figure 5:**
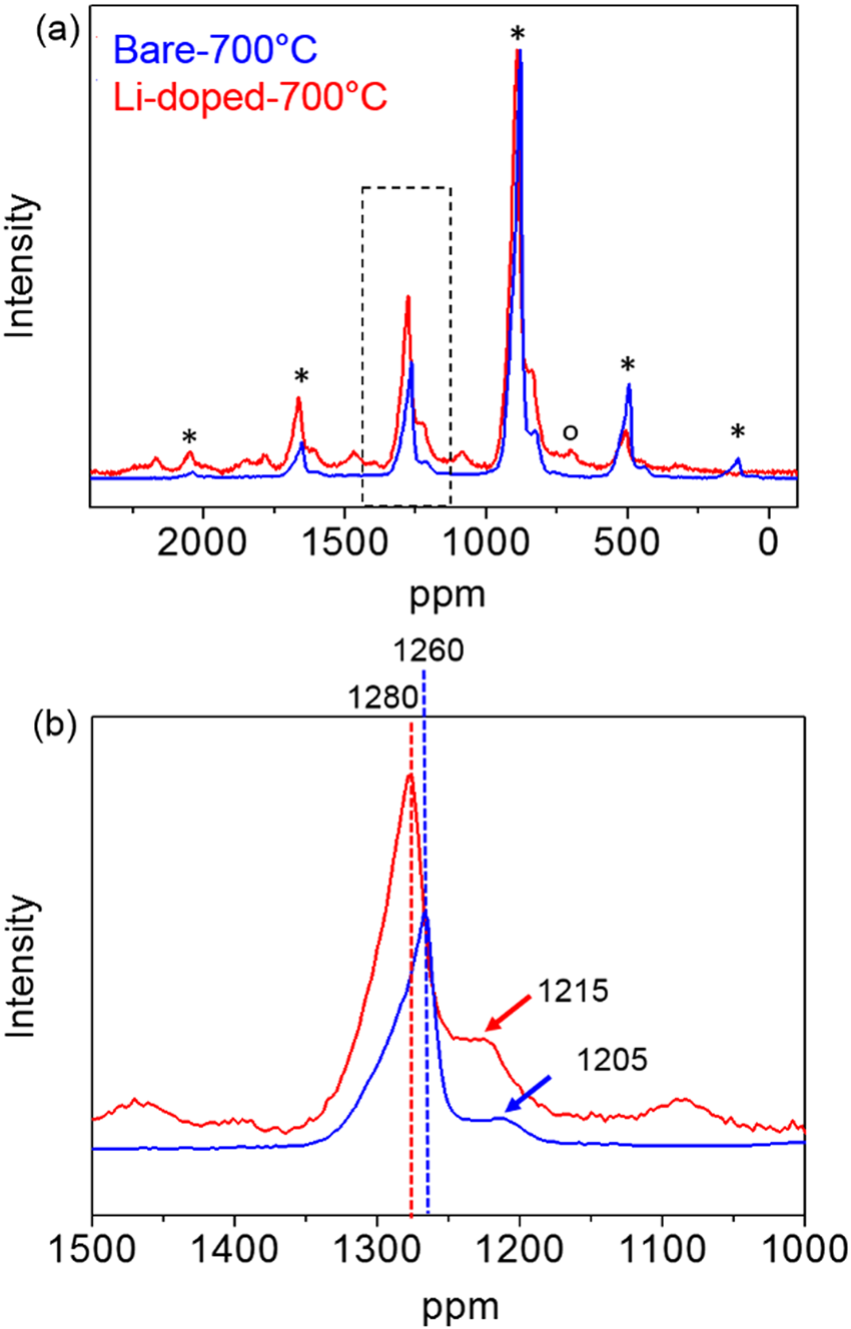
Comparison of ^7^Li MAS NMR spectra in (**a**) the bare −700 °C sample and the Li-doped −700 °C sample and (**b**) the comparison of ^7^Li MAS NMR spectra in lithium local environment in tetrahedral site in the two samples around 1200 ppm (asterisk*: sideband, circle(ο): signal of Li_2_MnO_3_).

To understand the effect of the Li doping on the degree of Ni/Mn ordering, the local Li environments in the two samples were investigated (Fig. 5). ^7^Li MAS NMR measurements were carried out on the samples with different spinning speeds to determine the isotropic resonances. Typically, the hyperfine shift of Li in high voltage spinel is caused by the oxidation state of surrounding Ni, Mn ions and the angle and the number of Li-O-Ni/Mn connections^12^.

The ^7^Li MAS NMR spectra of the two samples in Fig. 5 shows different Li local environments that can result from the different degree of Ni/Mn ordering and average oxidation state of Mn ions. In Fig. 5, at least two different Li local environments in the two samples can be observed. Firstly, a Li resonance at around 700~800 ppm (circle in Fig. 5a) in the Li-doped −700 °C sample can be assigned to Li in a Li_2_MnO_3_ secondary phase^31^. Secondly, a set of Li resonances at around 1200 ppm in the two samples can be attributed to Li in tetrahedral sites in the LNMO spinel structure. An early ^6^Li study by Grey *et al*. on a stoichiometric LiNi_0.5_Mn_0.5_O_4_ synthesized at 700 °C reported shifts around 950 ppm^28^. In a more recent study, resonances with larger shifts (between 900 and 1200 ppm) were observed for a more disordered LiNi_0.5_Mn_0.5_O_4_ spinel, synthesized at 900 °C, above the disordering transition^27^. The hyperfine shifts observed here are in good agreement with Li in tetrahedral sites in Ni/Mn spinel structure. The observed resonances are higher than previously seen for stoichiometric ordered LiNi_0.5_Mn_0.5_O_4_ although one major resonance can be observed at 1280 ppm and 1260 ppm for the Li-doped and bare spinel, respectively. Considering that in the ideal ordered spinel structure, Li in tetrahedral sites has 12 (9 Mn and 3 Ni) transition metals as first nearest neighbors with 115–120° angle connections, the most intense resonances at around 1280 ppm in Fig. 5b can be likely assigned to this configuration. The deviation of Li environments from the ideal one can explain the additional resonances. For instance, the introduction of cation disordering between the two transition metal sites can lead to new lithium environments originated from different Mn: Ni ratios in the first nearest neighbors. Typically, Mn^4+^ ions in the first coordination sphere of a tetrahedral Li ion in a spinel structure lead to larger shifts than Ni^2+^ ions. So the shoulder with a lower shift, between 1215 and 1250 ppm, can be assigned to Ni^2+^-richer environments while the resonances with higher shifts (between 1285 and 1300) can be assigned to Mn^4+^ richer environments. Also, the change of the oxidation state for Ni or Mn ions can strongly affect the Li local environment in tetrahedral sites resulting in different spectral features^12, 32^. Hence, the different Li local environments in tetrahedral sites in the samples indicate that the samples exhibit different partial Ni/Mn disordering or a slight change in the oxidation state of Ni or Mn ions.

In the two 700 °C samples, the Li local environments differ from each other. The spectra for both Mn-rich and Ni-rich environments in the Li-doped −700 °C sample shows higher hyperfine shift than those in the bare −700 °C sample: from 1260 ppm in the bare −700 °C sample to 1280 ppm in the Li-doped −700 °C sample for Mn-rich environment and from 1205 ppm in the bare −700 °C sample to 1215 ppm in the Li-doped −700 °C sample for Ni-rich environment in Fig. 5b. Considering that the bare −700 °C sample and the Li-doped −700 °C sample have similar Mn average oxidation state, the shift to the high frequency in the Li- doped −700 °C sample is not from the change in the average oxidation state of Mn. Furthermore, the Li-doped −700 °C sample shows slightly broader resonances than the bare −700 °C sample in Fig. 5b. Typically, the disordered spinel (bare-900 °C) structure has broader resonances than the ordered spinel (bare −700 °C) structure due to a wider distribution of local Li environments originated from different Ni and Mn distribution^9, 12, 32, 33^. Therefore, the wider-spanning set of resonances for the Li-doped −700 °C sample compared to the bare −700 °C sample can be related to a different degree of Ni/Mn ordering. It suggests that the Li-doped −700 °C sample has the increase of Ni/Mn disordering compared to the bare −700 °C sample. This NMR observation is consistent with structural, magnetic and electrochemical data.

#### Location of additional Li in Li-doped samples

**Figure 6:**
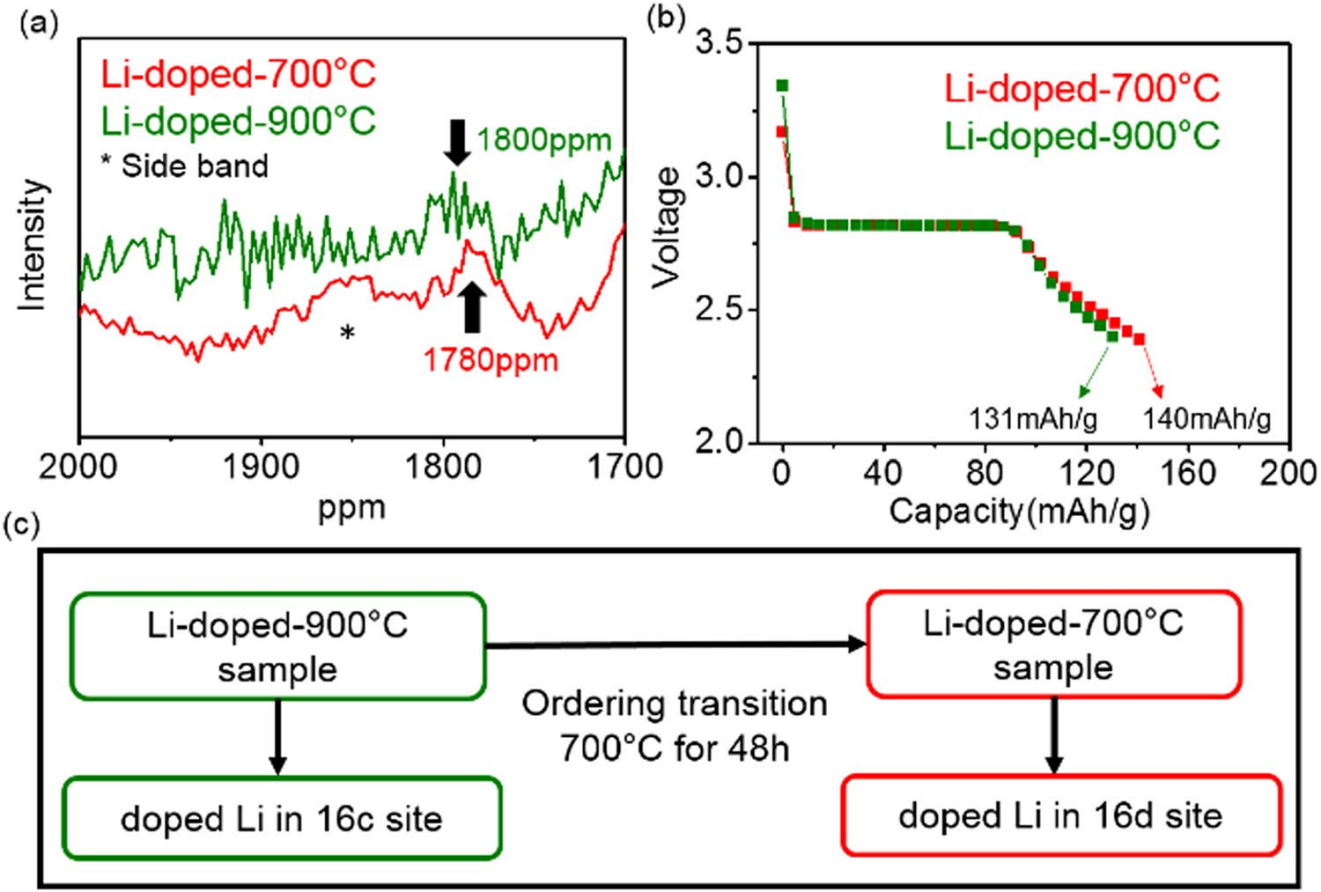
Comparison (**a**) ^7^Li MAS NMR spectra around 1800 ppm for the Li-doped-900 °C and Li-doped −700 °C sample (asterisk*: sideband) (**b**) Open circuit voltage (OCV) profiles below 3 V with discharging first of the two Li-doped samples (**c**) Schematic diagram of the occupation of the doped Li in the Li-doped samples during 2^nd^ annealing process at 700 °C.

NMR data in the Li-doped samples shows additional Li environment in octahedral site in addition to the two Li environments. In Fig. 6a, the additional resonance around 1800 ppm appears in the Li-doped samples and this resonance can be assigned to Li in octahedral site of the spinel^34^. The bare samples did not show any resonance between 1800 and 2300 ppm (in Figure S7). Therefore, this additional resonance indicates that some portion of the additional Li could be incorporated into the bulk of the spinel structure even though the Li-doped samples contains a trace of Li_2_MnO_3_ impurity. Taking available empty octahedral sites in the spinel structure into account, this additional Li resonance can be assigned to lithium in proximity to empty octahedral site (16c) in spinel structure or vacancy Ni site (16d in Fd3m or 4b in P4_3_32) associated with the deficiency of Ni set by the doping and higher oxidation state of Mn ions (Mn^4+^)^35^.

However, the additional Li local environment in the Li-doped −700 °C and Li-doped-900 °C sample is a little different. The resonance in the Li-doped −700 °C sample appears at ~1780 ppm whereas the resonance in the Li-doped-900 °C sample appears around 1800 ppm (Fig. 6a). This difference in resonance shift indicates that the local environment of the additional Li in the two Li-doped samples is different. During the annealing process (order - disorder transition) at 700 °C, the octahedral Li site is changed from ~1800 ppm in the Li-doped-900 °C sample to ~1780 ppm. Considering that the 16c site and 16d (4b in P4_3_32) site have different local coordination environment from different number of Li-O-M bonds and bond angles^35^, lithium in 16c site is generally observed higher hyperfine shift than 16d site^36, 37^. Therefore, the 16c site in the Li-doped-900 °C sample is dominantly occupied by additional Li ions whereas the 16d site in the Li-doped −700 °C sample is dominantly occupied. The Li doping in the Li-doped samples may move from 16c site in the Li-doped-900 °C sample to 16d site in the Li-doped −700 °C sample during 2^nd^ annealing process or the order-disorder transition process (Fig. 6c).

To further understand where the doping Li in the two Li-doped samples goes, we evaluated the electrochemical property of the two Li-doped samples below 3 V because the amount of capacity obtained below 3 V is well correlated with the amount of empty 16c site in the spinel structure^38^. As the amount of empty 16c site increases, the capacity obtained from below 3.0 V increases.

Capacities of the two Li-doped samples below 3 V were obtained by discharging first with GITT (Galvanostatic Intermittent Titration Technique) method in Fig. 6b. The Li-doped −700 °C sample has ~6% more capacity than the Li-doped-900 °C sample. This different capacity indicates that the Li-doped-900 °C sample has less amount of empty 16c site than the Li-doped −700 °C sample. But the Li-doped −700 °C sample has similar capacity below ~3 V with the bare −700 °C sample in Figure S8 indicating that the Li-doped −700 °C sample has similar amount of empty 16c site with the bare −700 °C sample. Therefore, 6% of lithium in 16c site in the Li-doped-900 °C sample may move to 16d (4b) site of the Li-doped −700 °C sample during re-annealing process at 700 °C. This movement can make the amount of 16c site in the Li-doped −700 °C sample similar with that in the bare −700 °C sample. This occupation of 16c site in the Li-doped-900 °C sample is in well agreement with NMR data in Fig. 6a. Furthermore, the amount of 6% lithium in 16d site in the Li-doped −700 °C sample is in good qualitative agreement with the amount of 5% vacancies in 16d site in the Li-doped samples that can be from 5% deficient Ni contents. And the amount of 6% doped Li in 16d site is almost consistent with refined occupancy results in Table 1. Therefore, some of the doped Li in the Li-doped-900 °C sample can go to 16c sites and the rest of it can form the secondary phase, Li_2_MnO_3_. During the order-disorder transition process at 700 °C, Li that sits at 16c sites in the Li-doped-900 °C sample can move to 16d (4b) sites in the Li-doped −700 °C sample.

The Li-doped −700 °C sample had greater Ni/Mn disordering than the bare −700 °C sample from structural, magnetic, and electrochemical analysis. This increase may be a result of the influence of additional Li environments in the Li-doped samples. For example, movement of Li from 16c sites to 16d (4b) sites during the order-disorder transition can affect the ordering of Ni/Mn in the ordered spinel because the movement can affect the ordering transition pathway that is related to the occupancy of 16c sites^24^. The occupancy of 16d (4b) sites by Li in the Li-doped −700 °C sample can also affect the Ni/Mn ordering due to the change of the electrostatic interaction with neighboring metals. Since the Li doping can affect both the order-disorder transition pathway and the electrostatic interactions between regular 16d (4b) sites at the same time, the doped Li in the spinel structure can be an effective way to increase the Ni/Mn disordering without the need to introduce Mn^3+^ ions.

## Discussion

### The Li doping can affect the order-disorder transition: it can block the Ni/Mn ordering transition pathway

**Figure 7:**
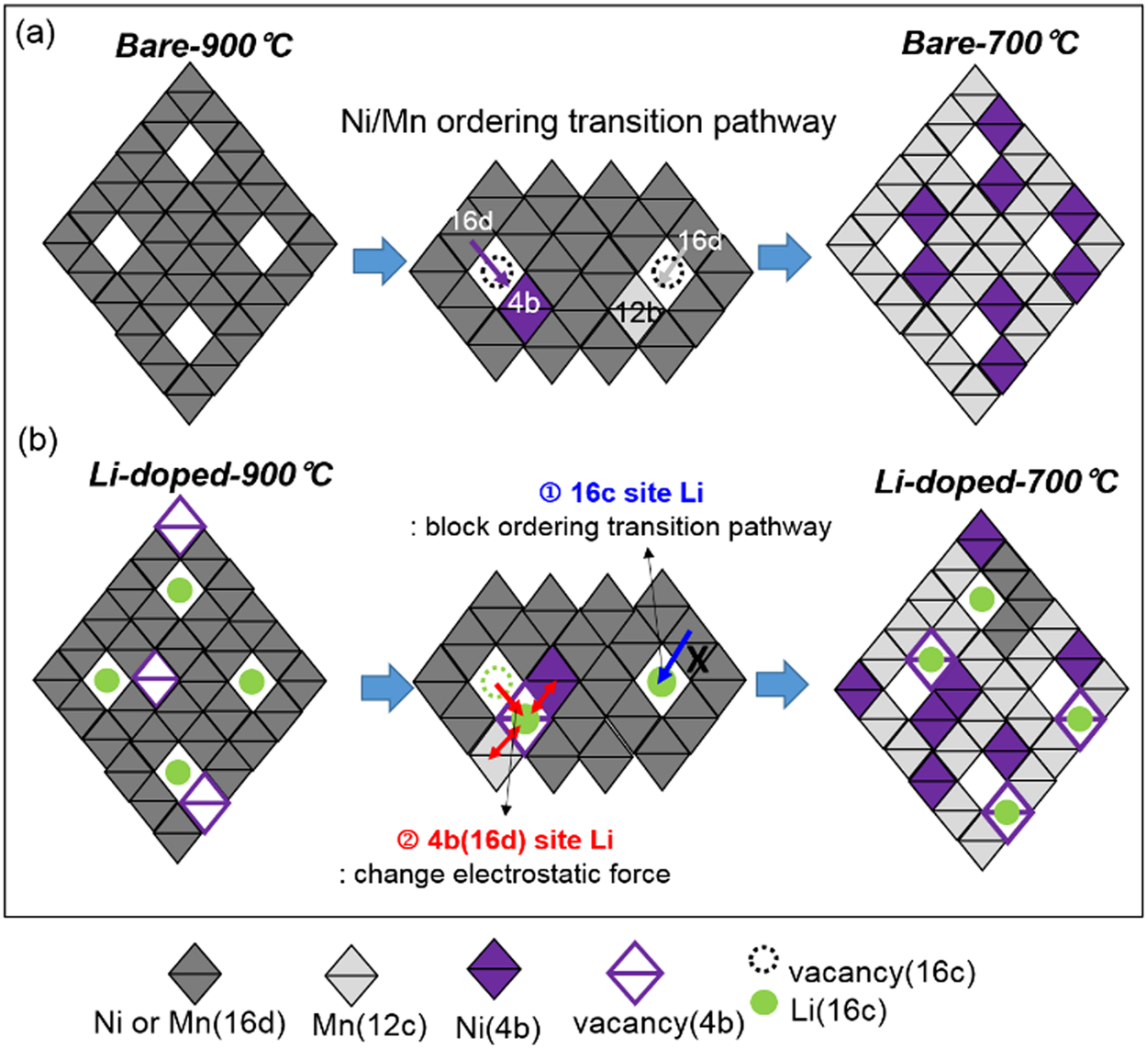
Schematic diagram of the Ni/Mn ordering transition pathway (**a**) bare sample (**b**) Li-doped sample (left: 900 °C structure, right: 700 °C structure).

The Li that was doped into the spinel was incorporated into the bulk. In the Li-doped-900 °C sample, the doped Li could occupy octahedral 16c sites, whereas in the Li-doped −700 °C sample it could occupy octahedral 16d (4b)) sites. As a result, the doped Li can move from 16c sites to 16d sites (4b sites in P4_3_32) during the order-disorder transition, and thereby affect not only the electrostatic interaction between Ni in 4b sites and Mn in 12c sites but also the order-disorder transition pathway. These two effects can increase the Ni/Mn disordering in the Li-doped −700 °C sample. Recent TEM measurement with DFT calculation^24^ shows that during the order-disorder transition in LNMO spinel the number of Mn/Ni atoms in 16d sites are displaced to the empty 16c sites to form sufficient vacancies in 16d sites; the presence of these vacancies facilitates the rearrangement of cation ordering (Fig. 7a). This transition pathway requires a sufficient number of atomic vacancies before Mn/Ni ions can be readily rearranged by vacancy migration to 16d sites (4b or 12c sites).

However, the Li-doped sample may have a different ordering mechanism because of the occupied Li at 16c and 16d sites. During the ordering transition in the Li-doped samples, Ni and Mn migrate from 16c to 16d sites and at the same time Li can migrate from 16c to 16d sites. This competition for sites by the doped Li affects the kinetics of the ordering transition. The effect of the doped Li on the Ni/Mn ordering transition can be understood as follows: the doped Li dominantly occupied empty 16c sites in the Li-doped-900 °C sample, and can therefore block the ordering transition pathway and thereby impede migration of Ni, Mn ions from 16d site to 16c site (Fig. 7b). This obstacle to the transition may result in incomplete ordering transition, and the increase in the Ni/Mn disordering in the Li-doped sample even after undergoing the ordering transition at 700 °C.

Furthermore, the doped Li in the Li-doped −700 °C sample could occupy 4b sites as shown in the NMR data. Considering that Ni^2+^ and Mn^4+^ ions occupy 4b and 12c sites, respectively, in the 700 °C spinel structure, the doped Li in 4b sites strongly perturbs electrostatic interactions between metals due to different oxidation state and different bond lengths compared to Ni/Mn. As a consequence, the Ni/Mn ordering in the Li-doped sample is less dominant. Therefore, the Li doping in the Li-doped −700 °C sample could increase the degree of Ni/Mn disordering by blocking Ni/Mn ordering transition pathway and perturbing the electrostatic interactions in 4b and 12c sites by occupying the regular Ni/Mn position even though the Li-doped −700 °C sample has experienced re-annealing process like the bare −700 °C sample.

The Li doping in LNMO can increase the Ni/Mn disordering even without the effect of Mn^3+^ ions. Even if the Li-doped sample does not show complete Ni/Mn disordering, which confirmed by superstructure in Neutron and TEM, the Ni/Mn ordering is suppressed; the increase in local cation disordering in spinel structure that is similar to cation disordering behavior in recent work on local structure in LNMO spinel^39^. In previous approaches, the disordering of Ni/Mn was achieved by adjusting the quantity of Mn^3+^ ions by introducing dopant ions into the spinel structure^21^ or by losing oxygen in the spinel at temperature process >700 °C or quenching processes^8, 20^. For example, the doping onto the Ni/Mn site has an effect on perturbing electrostatic interactions by randomly occupying the transition-metal sites. As a consequence, most of these previous approaches result in Ni/Mn disordering with Mn^3+^ ions even after re-annealing at 700 °C, but have little effect on the ordering transition pathway. The existence of Mn^3+^ ions in LNMO can have a negative effect on the electrochemical properties such as capacity retention and stability at high voltage due to the dissolution of Mn^15^.

Unlike these previous researches, a doping with Li to replace Ni can increase the Ni/Mn disordering without introducing Mn^3+^. The doped Li strongly affects electrostatic force due to the presence of Li in an Ni/Mn position, and the movement of Li from 16c to 16d (4b in P4_3_32) sites also affects the Ni/Mn ordering transition pathway. Therefore, the Li-doped −700 °C sample increased Ni/Mn disordering even after being subjected to Ni/Mn ordering transition during re-annealing. These findings demonstrate that the Ni/Mn disordering in LNMO spinel can be decoupled from the Mn^3+^ ions and be controlled by the Ni/Mn ordering transition pathway and changing the electrostatic interaction.

## Conclusion

The Li doping into high-voltage spinel increased the Ni/Mn disordering without the effect of Mn^3+^ ions on the disordering. The increase of Ni/Mn disordering was confirmed by Neutron diffraction, TEM image, broad voltage gap, and NMR data for different Li tetrahedral environments even though the sample underwent the ordering transition. Furthermore, the absence of Mn^3+^ ions in the Li- doped −700 °C sample was supported by the observation of negligible activity at ~4.0 V, and the similarity of lattice parameter to that of the bare −700 °C sample. NMR results show that the Li-doped samples have additional octahedral Li environments in additional to the Li tetrahedral environment that occur in the bare −700 °C sample; this result suggests that additional Li was incorporated into the bulk. Furthermore, NMR data for the Li-doped samples gives information on the sites where the doped Li can be found; Li occupied the 16c site in the Li-doped-900 °C sample but the 16d (4b) site in the Li-doped −700 °C sample. These occupied positions of the doped Li were confirmed by electrochemical evaluation at <3 V. Considering that the Li-doped −700 °C sample was prepared by annealing the Li-doped-900°C sample at 700 °C, the doped Li in the Li-doped samples can move from 16c sites to 16d(4b) sites in the Li-doped sample. This movement during the annealing process can interfere with the ordering transition pathway in the spinel: movement of the doped Li affects the Ni/Mn ordering transition pathway, and Li occupancy of Ni/Mn sites perturbs electrostatic interactions. These effects of the doped Li can cause increase of Ni/Mn disordering in the Li-doped −700 °C sample. Controlling the ordering transition pathway can be useful way to increase the Ni/Mn disordering that can improve electrochemical performance without a negative effect of Mn^3+^ ions.

## Methods

### Material synthesis

LiNi_0.5_Mn_1.5_O_4_ was synthesized by a solid-state reaction. Appropriate ratios of Li_2_CO_3_, MnO_2_, NiCO_3_ were ball-milled in acetone for 12 h. Bare sample had the ratio of Li:Ni:Mn = 1.0:0.5:1.5. And Li-doped samples had the ratio of Li:Ni:Mn = 1.1:0.45:1.5. Dried mix of precursors was pelletized and then calcined at 900 °C for 12 h in air. (Sample name: bare-900 °C, Li-doped-900 °C) After calcination, the pellets were ground, re-pelletized, and then annealed at 700 °C for 48 h in air^2^. (Sample name: bare −700 °C, Li-doped −700 °C)

### Material Characterizations

#### Neutron diffraction

Neutron powder diffraction (NPD) data were collected at room temperature on the high-resolution diffractometer Echidna at the OPAL facility (Lucas Height, Australia) using neutrons of wavelength 1.6220 Å. For the measurements, the samples of LiNi_0.5_Mn_1.5_O_4_ and Li_1.1_Ni_0.45_Mn_1.5_O_4_ in the form of ~1 g of powder were loaded in 6 mm diameter cylindrical vanadium cans. Rietveld analysis of the NPD data was performed using the Fullprof Suite with the default neutron scattering lengths.

#### NMR Measurements

^7^Li NMR measurements were carried out at room temperature on a Bruker Avance-200 spectrometer (B_0_ = 4.7 T, Larmor frequency v_0_ = 77.78 MHz in ^7^Li resonance). MAS spectra were obtained by using a Bruker MAS probe with a cylindrical 2.5-mm o.d. zirconia rotor. Spinning frequencies up to 34 kHz were used. ^7^Li MAS NMR spectra were acquired using a Hahn echo (π /2 – τ – π – τ) pulse sequence with a π/2 pulse of 2.3 µs. Recycle time was typically 0.5 s. The isotropic shifts, reported in parts per million (ppm), are relative to an external liquid 1 M solution of LiCl set at 0 ppm. Isotropic resonances were identified using variable spinning speed.

#### Magnetic property measurements

Magnetic susceptibility for the samples of LiNi_0.5_Mn_1.5_O_4_ and Li_1.1_Ni_0.45_Mn_1.5_O_4_ was measured using a vibrating sample magnetometer (VSM) technique on a Physical Property Measurement System (PPMS, Quantum Designs). The data were collected in the zero field cooled (ZFC) and field cooled (FC) modes with magnetic field of 500 Oe over the temperature range 2–300 K.

#### Electrochemical measurements

For the galvanostatic electrochemical test, the lithium metal half-cells were assembled using a Swagelok cell. The composite electrode weight ratio of [Active material 80 wt%: super-P (carbon black, Timcal) 5 wt%: carbon nano fiber 10 wt%: Binder 5 wt% (PVDF)] was prepared by spreading a slurry mixture on Al foil. The loading density of the electrode was 2–4 mg/cm^2^. The active cathode and a lithium metal anode were separated by a porous polypropylene film (Celgard 2400). Electrolyte used was 1 M LiPF_6_ dissolved in EC/DEC (1:1) solution. The cell was operated at room temperature.

## Electronic supplementary material

Supplementary information
