# Supplementary material for: Understanding the cation ordering transition in high-voltage spinel LiNi0.5Mn1.5O4 by doping Li instead of Ni
Source: Sci Rep. 2017 Jul 27;7:6728. doi: 10.1038/s41598-017-07139-2 (PMC5532243; doi:10.1038/s41598-017-07139-2)
Supplement: Supplementary file 1 — Supplementary information [file 41598_2017_7139_MOESM1_ESM.pdf]

Supporting information

Understanding the cation ordering transition in high-voltage spinel $LiNi_{0.5}Mn_{1.5}O_4$ by doping Li instead of Ni

Junghwa Lee [1], Nicolas Dupre [2], Maxim Avdeev [3], Byoungwoo Kang [1*]

[1]Department of Materials Science and Engineering, RIST 3131, Pohang University of Science and Technology (POSTECH),77 Cheongam-Ro. Nam-Gu. Pohang. Gyeongbuk. Korea (South) 790-784

[2] Institut des Materiaux Jean Rouxel (IMN), Universite de Nantes, CNRS, 2 rue de la Houssiniere, BP 32229, 44322 Nantes Cedex 3, France

[3]Australian Nuclear Science and Technology Organisation, Locked Bag 2001, Kirrawee DC, NSW 2232, Australia

[4] School of Chemistry, The University of Sydney, Sydney, NSW 2006, Australia

[*]E mail: bwkang@postech.ac.kr

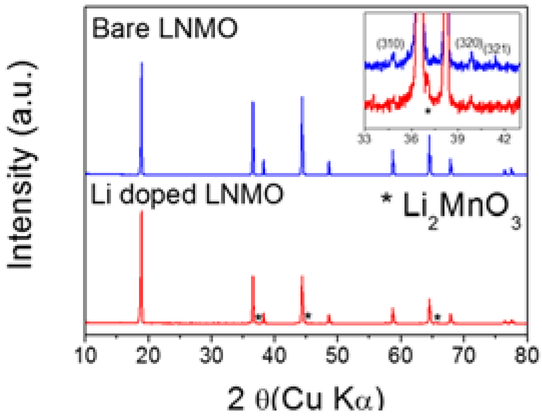

Figure S1. Synchrotron XRD patterns of (a) bare-700°C and Li-doped-700°C and comparison of the superstructure peak at (310), (320), (321) plane in both sample asterisk: signal of Li_2MnO_3[1]

Refined parameter		Sample name	
		Bare -700°C	Li-doped -700°C
Lattice parameter		8.171(1)	8.173(2)
Fractional coordination	Mn/Ni(12d)	(0.125,0.121,0.371)	(0.125,0.120,0.370)
	Ni/Mn(4b)	(0.375,0.375,0.375)	(0.375,0.375,0.375)
	Li(8c)	(0.754,0.754,0.754)	(0.754,0.754,0.754)
	O1(8c)	(0.134,0.134,0.134)	(0.134,0.134,0.134)
	O2(24e)	(0.125,0.350,0.357)	(0.126,0.351,0.357)
0.Occupancies	Mn/Ni(12d)	1.487/0.013	1.463/0.037
	Ni/Mn(4b)	0.487/0.013	0.430/0.037
	Li(4b)	0	0.067(3)
B_{iso}	Mn/Ni(12d)	0.354(60)	0.134(69)
	Ni/Mn(4b)	0.651(50)	0.319(61)
	Li(8c)	0.720(201)	0.689(264)
	O1(8c)	0.244(51)	0.266(54)
	O2(24e)	0.376(36)	0.338(27)
Agreement factors	R_p, %	7.96	8.42
	R_{wp}, %	8.39	9.30
	$\times^2$	3.28	4.75

Table S1. NPD data based Rietveld refinement results of the bare-700°C and the Li-doped-700°C (space group $P4_332$)

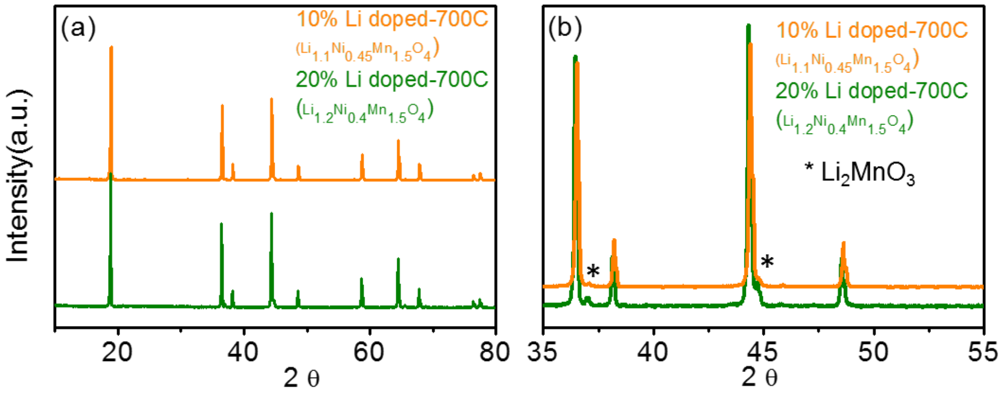

Figure S2. XRD patterns of 10% Li-doped-700℃ and 20% Li-doped-700℃ sample.

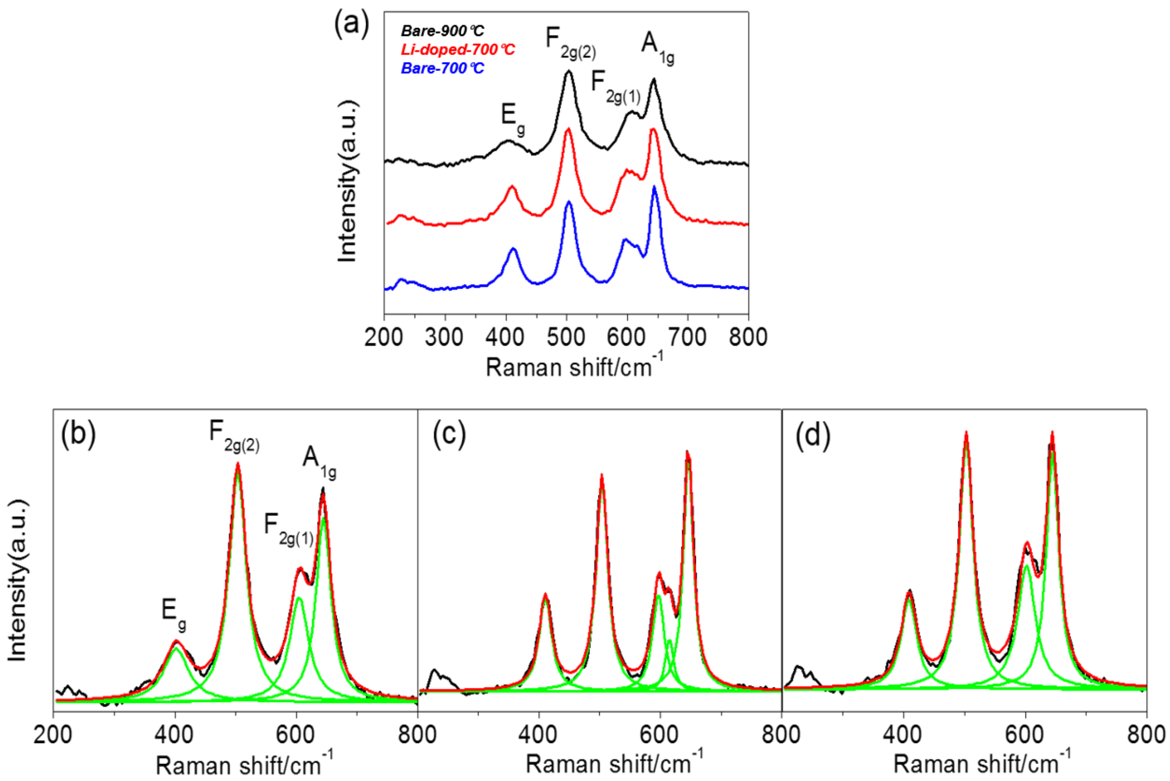

Figure S3. (a) Raman spectra of three different LNMO spinel sample. Deconvolution Raman spectra of (b) bare-900°C sample (c) bare-700°C sample and (d) Li-doped-700°C sample

Peak designation	FWHM		
	(a) Bare-900°C	(b) Bare-700°C	(c) Li-doped-700°C
A_{1g}	29.6827	19.4846	24.8280
$F_{2g(1)}$	38.1104	22.2156	35.2368
$F_{2g(2)}$	34.3361	23.1244	27.5320
E_g	50.0178	24.8865	30.7332

Table S2. Peak designation and FWHM of deconvoluted Raman spectra of the (a) bare-900°C sample (b) bare-700°C sample and (c) Li-doped-700°C sample

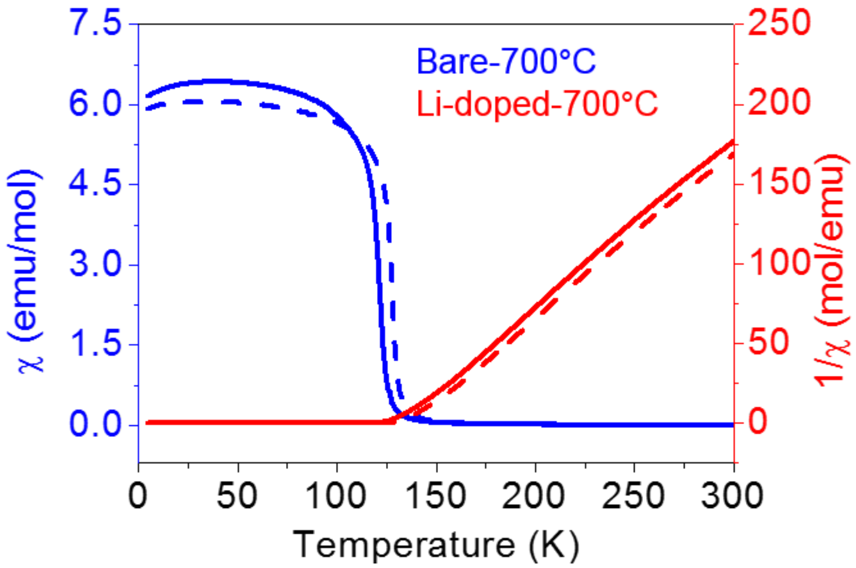

Figure S4. Temperature dependences of a magnetic susceptibility (left axis) and reciprocal magnetic susceptibility (right axis) for both samples. Magnetic susceptibility was measured in FC mode with H = 107/4 πA m^{-1}.

sample	T_C	μ_{eff}
(a) Bare-700°C	128.09	3.001
(b) Li-doped-700°C	121.90	3.030

Table S3. Ferrimagnetic ordering transition (T_c) and effective magnetic moment (μ_{eff}) (a) bare-700°C sample and (b) Li-doped-700°C sample

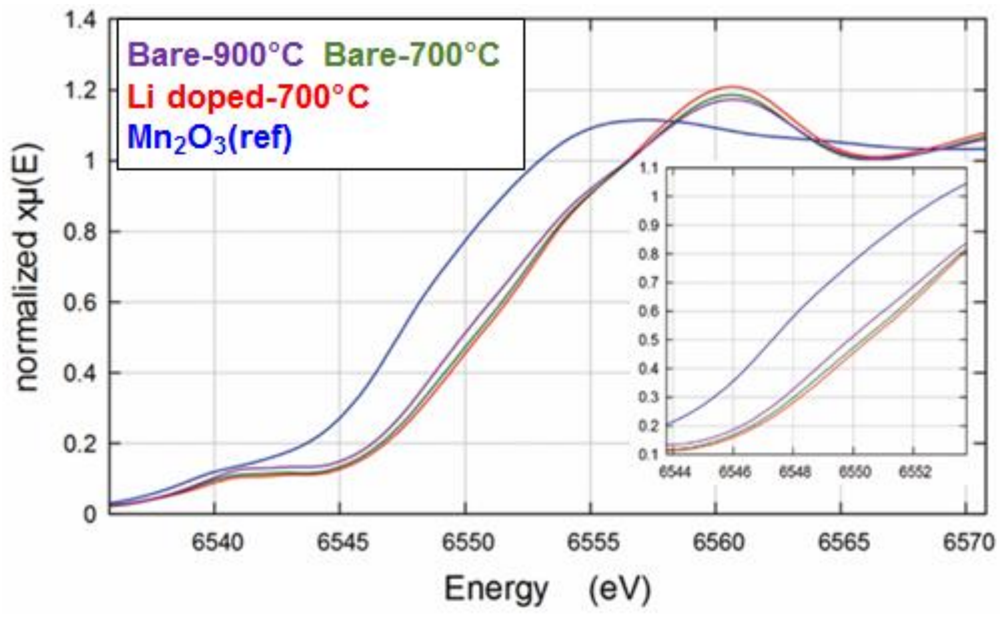

Figure S5. Mn K-edge XANES data for the bare-700°C sample (green straight line), the Li doped-700°C sample (red straight line), the bare-900°C sample (purple straight line) and Mn_2O_3 reference (blue straight line)[1]

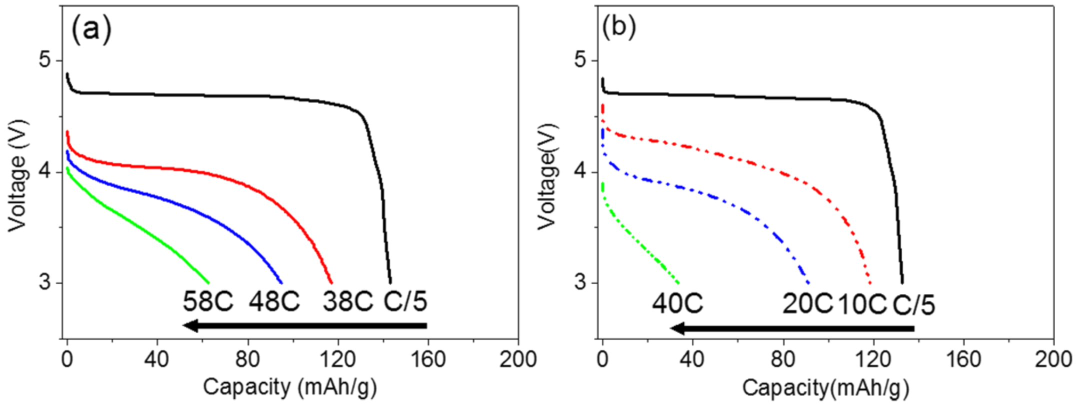

Figure S6. Rate capability curve of (a) Li-doped-700°C sample[1] and (b) bare-700°C sample without the voltage hold at the end of charge. Charge rate was C/5 without holding the voltage and discharge rates were different. Cutoff voltages were set from 3 to 5V. [1]

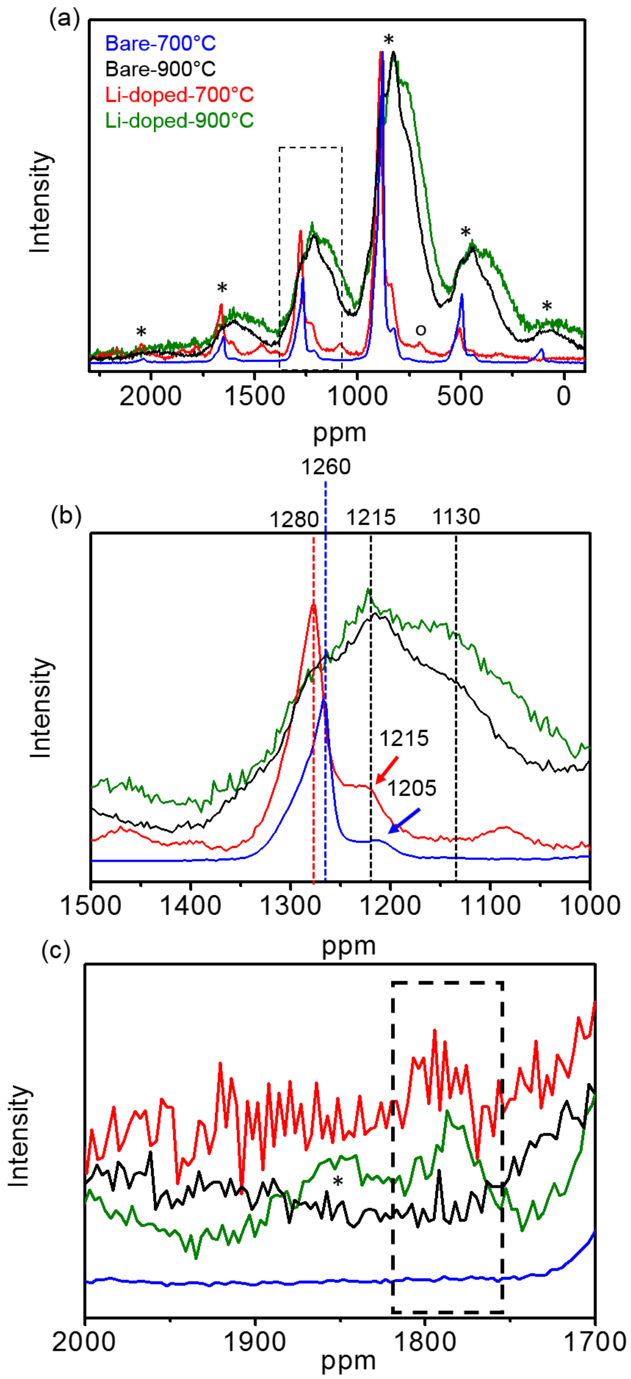

Figure S7. Comparison ^{7}Li MAS NMR spectra (a) bare 700°C /900°C samples and Li-doped 700°C /900°C samples and comparison ^{7}Li MAS NMR spectra of (b) lithium local environment in tetrahedral site of spinel around 1200 ppm (c) additional lithium in octahedral site for Li-doped-900°C and Li-doped-700°C LNMO around 1800 ppm (asterisk (*): sideband, circle (o): signal of Li_2MnO_3)

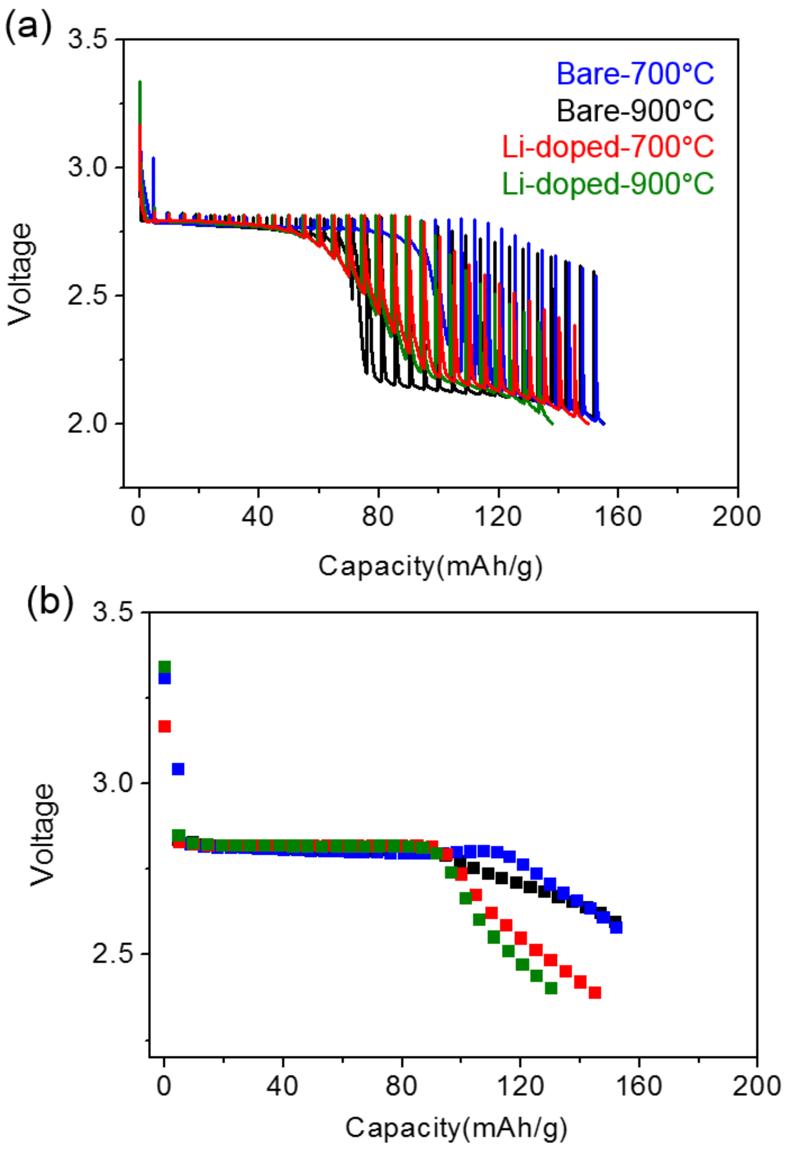

Figure S8. Comparison (a) GITT data (b) open circuit voltage (OCV) profiles below 3V with discharging first of bare LNMO and Li-doped LNMO samples

Reference

1 Junghwa Lee, C. K. a. B. K. High electrochemical performance of high-voltage $LiNi_{0.5}Mn_{1.5}O_4$ by decoupling the Ni/Mn disordering from the presence of Mn^{3+} ions. *NPG Asia Materials* **7**, e211 (2015).
